# Characterization of the NAC gene family in ‘Fengdan’ peony (*Paeonia ostii*) insights into the evolution and expression patterns under abiotic stresses and ABA treatment

**DOI:** 10.3389/fpls.2025.1559667

**Published:** 2025-04-16

**Authors:** Xiangli Yu, Qirui Meng, Hongyan Hou, Qiang Guo, Qingjie Wang, Yuqing Yang, Yanzhao Zhang

**Affiliations:** ^1^ School of Life Science, Luoyang Normal University, Luoyang, Henan, China; ^2^ School of Chemical Engineering, Huaqiao University, Xiamen, Fujian, China

**Keywords:** ‘Fengdan’ peony, NAC transcription factors, bioinformatics, abiotic stresses and ABA treatment, expression analysis

## Abstract

**Background:**

As one of the largest plant-specific transcription factor families, NAC proteins are crucial for plant growth and development processes and responses to various abiotic and biotic stresses. The published sequenced chromosome-level genome of ‘Fengdan’peony provides a powerful tool for the analysis of the NAC gene family in this shrub.

**Methods:**

The *PoNAC* gene family was identified and characterized using bioinformatic analysis, and RT-qPCR analysis was performed on some PoNACs from the ATAF and NAP subfamilies.

**Results:**

In this study, a total of 82 NAC transcription factors (TFs) were identified in the ‘Fengdan’ peony genome, with the uneven anchorage of 78 *PoNAC* genes on 5 chromosomes, whereas only 4 *PoNAC* genes were found to be located on unanchored scaffolds. Through the phylogenetic analysis, 66 *PoNAC* genes were classified into 15 distinct subfamilies. The gene structure analysis revealed the variation in the number of exons from 0 to 14. Moreover, the motif analysis indicated that the identified PoNAC TFs possessed conserved NAC domains and motifs. The duplication events of *PoNAC* genes included whole-genome duplications (WGDs) or segmental duplications for 14 pairs, tandem duplications for 2 pairs, and proximal duplications for 3 pairs. GO analysis results suggested that the functions of *PoNAC* genes were mostly concentrated in the “biological process” GO category. Additionally, the analysis of the expression profiles of *PoNAC* genes in different plant organs revealed that only 45 genes were expressed in various tissues, some of them exhibited tissue-specific expression related to plant growth and development. RT-qPCR experiments demonstrated the responses of 8 genes from the ATAF and NAP subfamilies to ABA, heat and drought, suggesting that they may serve as important regulatory factor.

## Introduction

During their growth and development, plants encounter both biotic and abiotic stressors, which directly impact these processes as well as their yield. To overcome these challenges, plants have evolved complex regulatory networks over the course of their long-term evolution, which not only play a crucial role in the growth and development, but also have significant importance for plants in coping with stress responses (Zuo et al., 2023). Transcription factors (TF) regulate the expression of genes by either activation or inhibition through binding to specific DNA sequences located in the upstream promoter region of their target genes. The NAC TF family constitutes one of the largest and most widely distributed transcription factor families across the plant kingdom. Its proteins share a common sequence in the NAC domains (Ooak et al., 2003), including *no apical meristem* (NAM; in glory) (Duval et al., 2002), *ATAF1/2* (in Arabidopsis) (Christianson et al., 2010), and *cup-shaped cotyledon* (*CUC2*; in Arabidopsis) (Aida et al., 1997).

Apart from a highly variable C-terminal transcriptional regulatory region (TRR), members of the NAC gene family possess a highly conserved N-terminal domain consisting of around 150 amino acids, which can be further separated into five subdomains (A to E) (Singh et al., 2021). Among them, subdomain A is involved in the formation of functional dimers, while B and E subdomains drive the functional diversity of *NAC* genes, and C and D subdomains, which include nuclear localization signals perform the DNA-binding activity (Jia et al., 2019). Although the NAC domain of different plants shares structural similarities, it has different functions in various plant parts and under different stress factors. The roles that *NAC* genes play in plants comprise the development of the whole plant, leaves (Hasson et al., 2011), and lateral roots (Xie et al., 2000), meristem maintenance (Zhan et al., 2021), secondary cell wall synthesis (Mccarthy et al., 2011), leaf senescence (Wang et al., 2021; Melo et al., 2021), etc.

The overexpression of *ANACO32* in *Arabidopsis* can cause the downregulation of the expression of some anthocyanin biosynthesis genes, including *DFR*, *ANS*, and *LODX*, under stress conditions, which results in the regulation of anthocyanin biosynthesis (Meraj et al., 2020). Under drought stress, the survival rate and antioxidant enzyme activity of Arabidopsis plants overexpressing the maize *ZmSNAC06-T02* gene were both higher than those of the wild type (Wang et al., 2025), while *GmNAC20* in soybean can improve the tolerance of rice to cold stress (Yarra and Wei, 2021). Moreover, *CaNAC2c* can perform various functions in pepper, including the activation of the heat shock transcription factor *CaHSFA5*, prevention of the accumulation of H_2_O_2_, and enhancement of heat stress tolerance in the plant (Cai et al., 2021), and the arabidopsis *ataf1* mutant was found to have higher survival rate and fresh weight than its wild-type counterpart under heat stress (Alshareef et al., 2022).

‘Fengdan’ peony (*Paeonia ostii*) is a traditionally famous flower native to China (Sheng et al., 2023) whose ornamental, medicinal, and oil values (Yang et al., 2020; Wang et al., 2019) have driven the rapid expansion and development of its seedling industry in recent years (Guo et al., 2023). Tree peonies often experience environmental stress factors such as heat, drought and low temperature during their growth and development, which may consequently be negatively affected and even lead to plant death. Therefore, the mechanisms of stress resistance in tree peonies and the cultivation of their stress-resistant varieties have attracted increasing attention from researchers (Yang et al., 2024; Ma et al., 2022; Gai et al., 2024; Xu et al., 2017). Due to its paramount importance in various developmental processes and stress responses in tree peonies, studying the NAC gene family in tree peonies is crucial. In this study, members of NAC transcription factor family were identified and performed bioinformatic analyses based on the annotated genome of “Fengdan” peony. Furthermore, “Fengdan” peony was subjected to drought stress, hot stress, and ABA treatments, with RT-qPCR expression analysis conducted on 8 selected *PoNAC* genes. The results provide insights into the structure and function of *PoNAC* genes.

## Materials and methods

### Plant materials and treatments

Three-year-old healthy seedlings of ‘Fengdan’ peony with uniform size without any pest and disease symptoms, grown on the campus of Luoyang Normal University, were transplanted into 30-L pots filled with soil in winter. The potted tree peonies were first placed under natural conditions and then transferred to an indoor plant growth room under a 14-h photoperiod at 25°C, with a light intensity of 25 μmol m^-2^s^-1^ and relative humidity of 70%. The application of the ABA treatment and heat and drought stresses was performed separately. Upon the maturity of leaves, plants were placed in the incubator at 42°C to impose heat treatment for 45 days, while the 100μmol/L ABA solution and 20% PEG 6000 solution were sprayed on the leaves of plants in the thermostatic growth room to perform the ABA treatment and simulate drought stress, respectively, and control plants were sprayed with ddH_2_O. First, leaves were collected at 0 h, 3 h, 6 h, 12 h, and 24 h after the treatments, frozen in liquid nitrogen, and finally stored at –80°C for the subsequent extraction of total RNA and analysis of gene expression. The Total RNA Extraction Kit (Lingjun, Shanghai, China) was used to extract total RNA from ‘Fengdan’peony plants, which was then reverse-transcribed into cDNA using PrimeScript RT Reagent Kit, a reverse transcription kit (TaKaRa, Dalian, China) for a RT-qPCR assay.

### Genome-wide identification of NAC gene family members in ‘Fengdan’peony and prediction of protein subcellular localization

All ‘Fengdan’peony genome data were obtained from the China National GeneBank DataBase (CNGBdb) (https:db.cngb.org/search/assembly/CAN0050666), while the genomic data for *Arabidopsis thaliana* were derived from the Arabidopsis Information Resource (TAIR) (https://www.arabidopsis.org/). The Hidden Markov Model (HMM) profile of the NAM domain (PF02365) was retrieved from the InterPro database (https://www.ebi.ac.uk/interpro/) to identify *NAC* genes from the ‘Fengdan’ peony genome using the HMMER 3.3.2 software, and thereafter, the generated candidate proteins were filtered based on a score value of higher than 50 and an E-value of lower than 1 × 10^−5^ by NCBI Conserved Domain Search (CD-Search) (https://www.ncbi.nlm.nih.gov/Structure/bwrpsb/bwrpsb.cgi) and SMART (http://smart.embl-heidelberg.de). To mine the NAC family members of ‘Fengdan’ peony, the proteins without the NAM domains and repeats were manually deleted. The ExPASy ProtParam tool (http://web.expasy.org/protparam/) (Gasteiger et al., 2003) and WoLF PSORT (https://wolfpsort.hgc.jp/) (Horton et al., 2006) were used to predict the subcellular localization and physical and chemical properties of the identified NAC proteins.

### Analysis of chromosomal localization, duplication events, and Ka/Ks of NAC gene family members in ‘Fengdan’ peony

Data on the chromosomal location of *NAC* genes were obtained from the genome GFF3 file of ‘Fengdan’ peony. TBtools software (Chen et al., 2023) was used for the analysis of duplication events of *NAC* genes in ‘Fengdan’ peony and visual display. To calculate the non-synonymous (Ka) and synonymous (Ks) substitution rates for duplicated genes, KaKs_Calculator (Zhang, 2022) was used.

### Phylogenetic analysis of the NAC gene family

To further explore the phylogenetic relationships among ‘Fengdan’ peony NAC gene family members, the phylogenetic tree of NAC proteins from *P. ostii* and *A. thaliana* was constructed in the MEGA-11.0 software (Sudhir et al., 2018) using the neighbor-joining (NJ) method with 1000 bootstrap replicates and then edited with the Interactive Tree of Life online tool (iTOL; https://itol.embl.de/). All PoNAC proteins were classified according to the branching and classification standards of ANAC proteins from Arabidopsis (Ooak et al., 2003).

### Analysis of the gene structure, conserved motifs, and conserved domains of the NAC gene family

To further clarify the evolutionary relationships among PoNAC gene family members, and predict the conserved sequence of NAC proteins in ‘Fengdan’ peony, the MEME online tool ( https://meme-suite.org/meme/tools/meme ) (Timothy et al., 2015) with 15 motifs was used, and the Hit Data File was obtained from the NCBI Batch CD-Search (http://www.ncbi.nih.gov) (Marchler-Bauer et al., 2015), while the NAC gene structure in ‘Fengdan’ peony was analyzed using the organized genome GFF3 file. Tbtools was used to visualize the conserved motifs, conserved domains, and gene structure (Chen et al., 2023).

### Gene ontology enrichment analysis

Gene ontology (GO) functional annotation of the *PoNAC* genes was performed based on three distinct aspects, including cellular component, molecular function, and biological process using the WEGO online tool (https://wego.genomics.cn/) (Ye et al., 2018).

### Analysis of transcript abundance of the NAC gene family in different tissues of ‘Fengdan’ peony

The raw data of the expression of *NAC* genes in various tissues of tree peony, such as seeds, leaves, and buds were explored based on the genomic data from previous studies (Zhang et al., 2020). Thereafter, to present the results, a heatmap was generated by TBtools using the gene log2 ^(FPKM+1)^ values, which represent the gene expression levels (Chen et al., 2023).

### RT-qPCR analysis

The primers presented in **Supplementary Table 1** were designed using the GenScript online website (https://www.genscript.com/tools/real-time-pcr-taqman-primer-design-tool), while the analysis of gene expression by RT-qPCR was performed with three technical replicates using ubiquitin (Wang et al., 2012) as an appropriate reference gene. Each qPCR reaction (20 µl) containing 10 µl TB Green Premix Ex Taq II (2×), 2µl cDNA as template, 0.5 µl forward primer, 0.5 µl reverse primers, and 7 µl nuclease-free water was run under the conditions and according to the procedures described in previous studies (Guo X, et al., 2024). To quantify the expression levels of *PoNAC* genes, the 2^−ΔΔCt^ method was used, and data were analyzed with analysis of variance (ANOVA) using SPSS 26.0 and visualized with Graphpad Prism 9.3.

## Results

### Identification of PoNAC genes in ‘Fengdan’ peony

In this study, based on the whole-genome data of ‘Fengdan’ peony, 82 NAC proteins were identified and then named *PoNAC1*-*PoNAC82* according to the number of gene sequences, and their characteristics are provided in **Supplementary Table 2**. These proteins encoded by the *PoNAC* genes contained amino acids within the range of 110 (*PoNAC32*) to 726 (*PoNAC5*), and their molecular weights (MW) ranged from 12.72 (*PoNAC5*) to 83.09 KDa (*PoNAC32*), with an average value of 39.71 KDa. The isoelectric points (pI) of these proteins varied from 4.75 (*PoNAC21*) to 9.99 (*PoNAC25*) (average value of 7.22). Except for *PoNAC31*, all PoNAC proteins had the negative grand average of hydropathicity index (GRAVY), indicating that they were hydrophilic proteins. Furthermore, the prediction of subcellular localization of these proteins revealed that they were mostly present in the nucleus (46), followed by cytoplasm (15) and chloroplast (10).

### Chromosome mapping and analysis of duplication events and evolutionary selection pressure of *PoNAC*s

**Figure 1:**
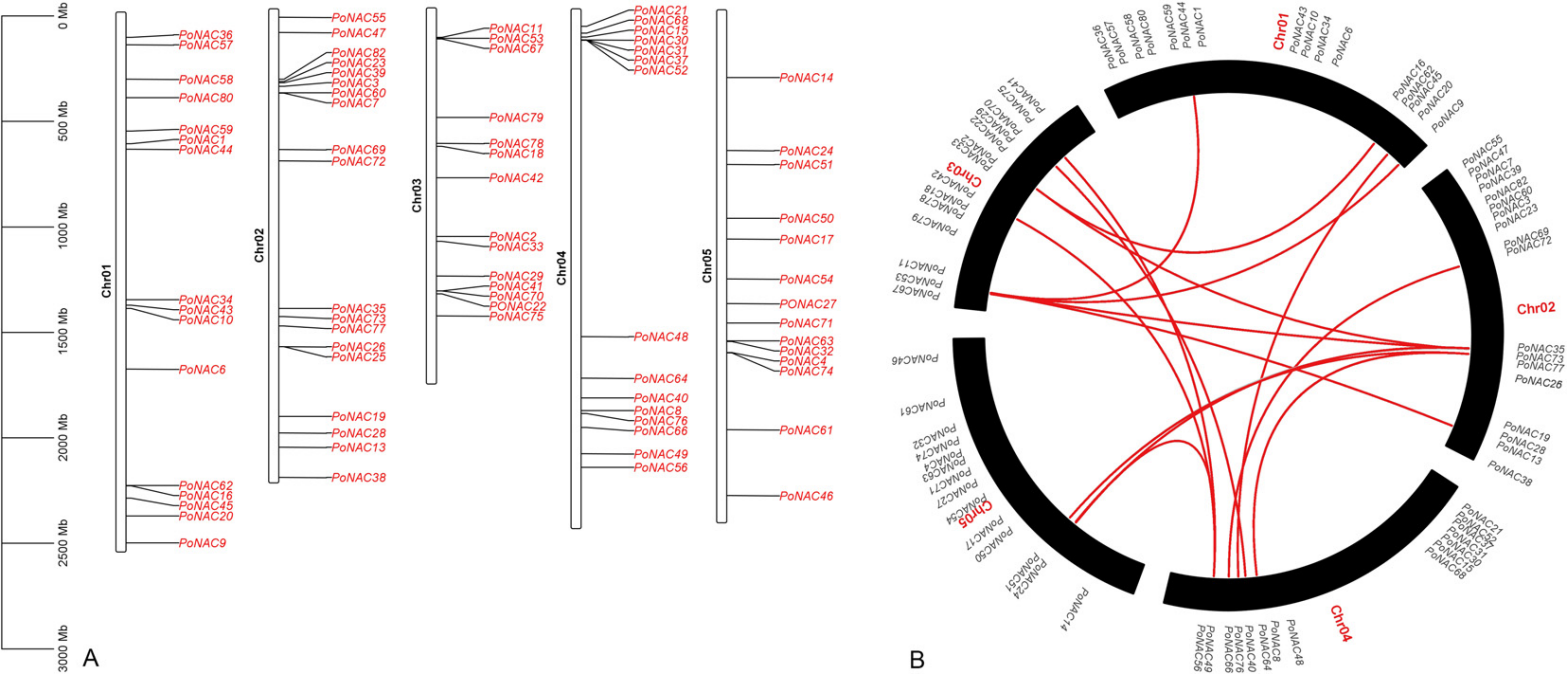
Chromosomal distribution and duplication events of *PoNAC* genes in ‘Fengdan’ peony. **(A)** Localization of *PoNAC* genes on chromosomes. The scale bar on the left indicates the length (Mb) of chromosomes **(B)** Duplication events of *PoNAC* genes. The red line in the circle indicates the collinearity gene pair.

A total of 4 genes (*PoNAC5*, *PoNAC65*, *PoNAC12*, and *PoNAC81*) were found to be located on unchr_scaffold_974, unchr_scaffold_5036, and unchr_scaffold_2044. **Figure 1A** shows the distribution of 78 *PoNAC* genes on different chromosomes. The chromosomes 1, 2, 3, 4, and 5 contained 16 (20.51%), 19 (24.3%), 14 (17.95%), 15 (19.23%), and 14 (17.95%) *PoNAC* genes, respectively, which were mainly distributed at both ends, but less in the middle of each chromosome.

To facilitate the analysis of gene duplication events in tree peonies, all unchr_scaffolds were deleted from the ‘Fengdan’ peony genome, and only the information carried in 5 chromosomes was retained (**Figure 1B**). A total of 19 pairs of gene duplication events were detected in ‘Fengdan’ peony during its evolution, of which 14 pairs underwent whole-genome or segmental duplication, while 2 pairs(*PoNAC12-PoNAC81* and *PoNAC31-PoNAC37*) were tandemly duplicated genes, and 3 pairs including *PoNAC25-PoNAC26*, *PoNAC30-PoNAC52*, and *PoNAC41-PoNAC70* experienced proximal duplication.

The selection pressure of genes undergoing duplication events was estimated based on the Ka (non-identical)/Ks (identical) values, which were calculated using the Ka/Ks calculator software (**Supplementary Table 3**). The Ks values of *PoNAC* gene pairs ranged from 0.00966144 to 4.02808, with 15 pairs (78.9%) having Ks values of above 1. The Ka/Ks values of 18 pairs (94,7%), however, were below 1, indicating the evolution of these genes under strong purifying selection. Only 1 pair (Ka/Ks > 1) might have undergone evolution and functional changes under positive selection after duplication.

### Analysis of phylogenetic relationships of PoNAC gene family members in ‘Fengdan’ peony

**Figure 2:**
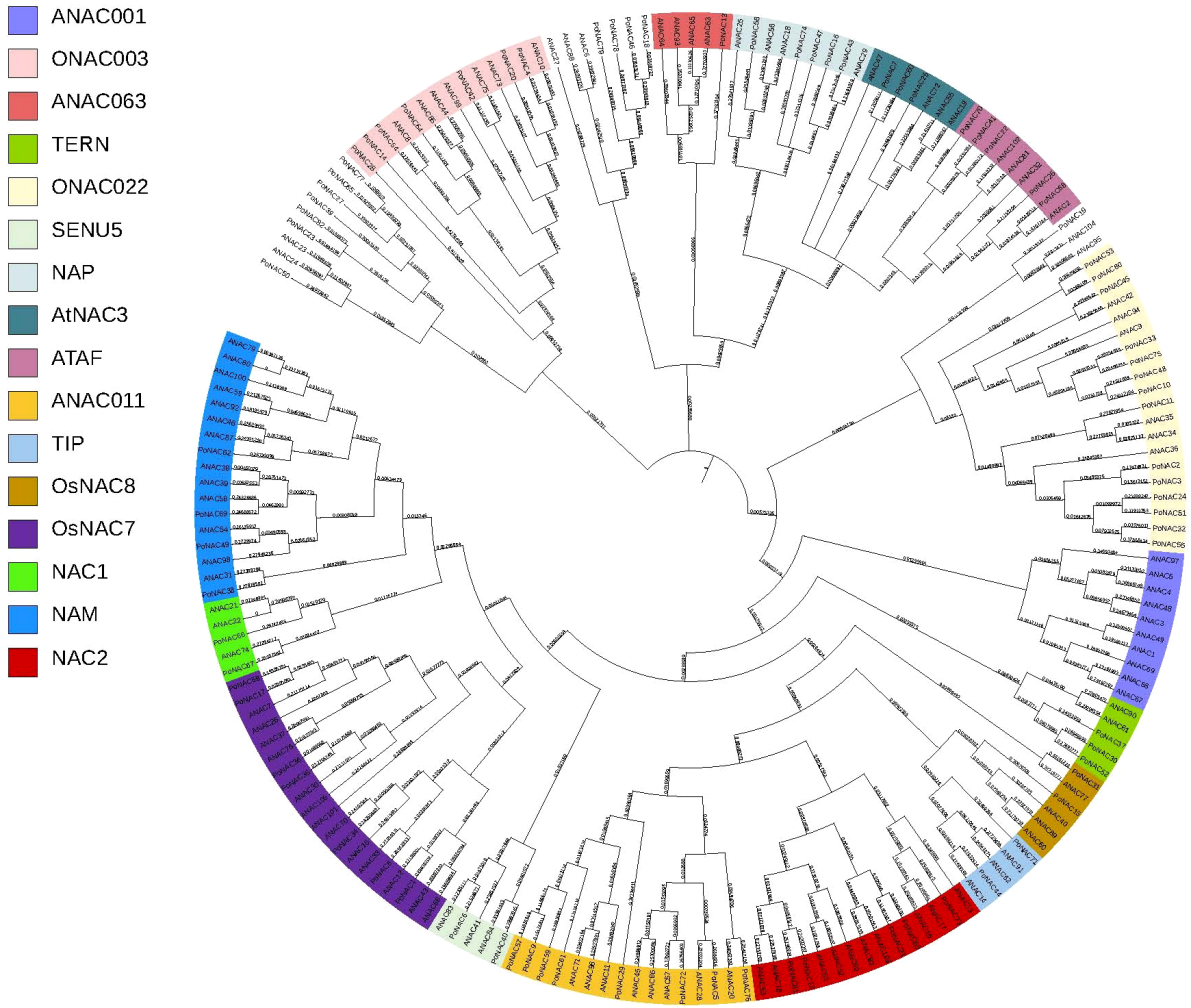
A phylogenetic tree of *NAC* proteins from ‘Fengdan’peony and Arabidopsis. The colors on the left represent different subfamilies.

The phylogenetic tree was constructed by combining 82 proteins from ‘Fengdan’ peony with 105 ANAC proteins from Arabidopsis using MEGA11.0 software (**Figure 2**). These 82 *PoNACs* in ‘Fengdan’ peony were further divided into 15 subfamilies including ONAC003 (7), ANAC063 (1), TERN (3), ONAC022 (14), SENU5 (2), NAP (5), ANAC3 (3), ATAF (5), NAC2 (5), ANAC011 (8), TIP (2), OsNAC8 (2), OsNAC7 (7), NAC1 (2), and NAM (4) according to the phylogenetic tree. However, the ANAC001 subfamily contained no ‘Fengdan’ peony PoNACs. Since PoNAC protein sequences and unclassified protein sequences from Arabidopsis were phylogenetically clustered, 16 PoNAC protein sequences could not be classified into subfamilies temporarily. **Figure 2** shows some specific NAC subfamilies in both Arabidopsis and ‘Fengdan’ peony, most of which are common among the two.

### The gene structure and motif analysis of *PoNAC* genes

**Figure 3:**
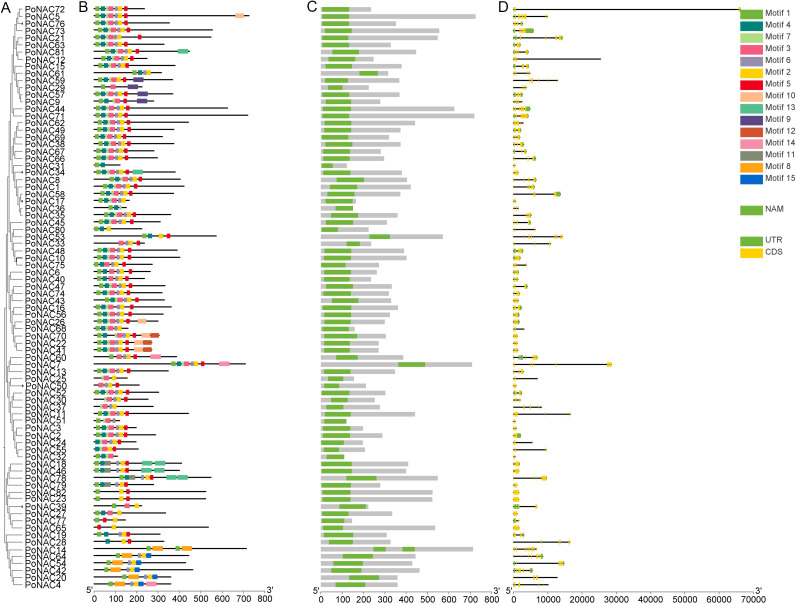
Classification, conserved motifs, and gene structure analysis of *PoNAC.* **(A)** Phylogenetic relationship of PoNAC based on NJ method. **(B)** Motif distribution of PoNAC proteins, different motifs are represented by different colors, ranging from1 to 15. **(C)** Conserved domain organization in PoNAC proteins. **(D)** Exon-intron structures of *PoNAC* genes.

The analysis of PoNAC gene family members’ conserved motifs, domains, and exon-intron structures was performed. A phylogenetic tree was reconstructed from evolutionary data using the neighbor-joining (NJ) method based on the multiple sequence alignment for the PoNAC gene family (**Figure 3A**). A total of 15 motifs representing the structural similarity and diversity of NAC proteins in ‘Fengdan’ peony were discovered using the MEME program (**Figure 3B**). The length of these conserved motifs varied from 11 to 50 amino acids, among which at least 2-8 were present in all 82 PoNAC proteins, and these motifs and phylogenetic relationships showed consistent results. Members having similar conserved motifs grouped in the same phylogenetic clades may also share similar functions. Moreover, all PoNAC proteins contained a conserved NAM domain, which is a specific conserved domain of the NAC gene family, indicating the reliable identification of the genes (**Figure 3C**). To further examine the structural characteristics of the NAC gene family in tree peonies, the distribution of exons and introns per *PoNAC* gene was visualized (**Figure 3D**). All *PoNAC* genes had a number of introns varied from 0 to 14, and gene members within the same subfamily exhibited a similar number of introns. Among them, ONAC003 subfamily members had 4-6 introns, while NAP subfamily members possessed 2 introns, and 1-2 introns were present in ATAF subfamily members. The existence of introns causes the structural diversity of *NAC* genes and their functional diversification. The analysis results of phylogenetic relationships, conserved motifs, conserved domains, and gene structure indicated that *PoNACs* remained highly conserved throughout the long evolutionary process.

### Gene ontology annotation

**Figure 4:**
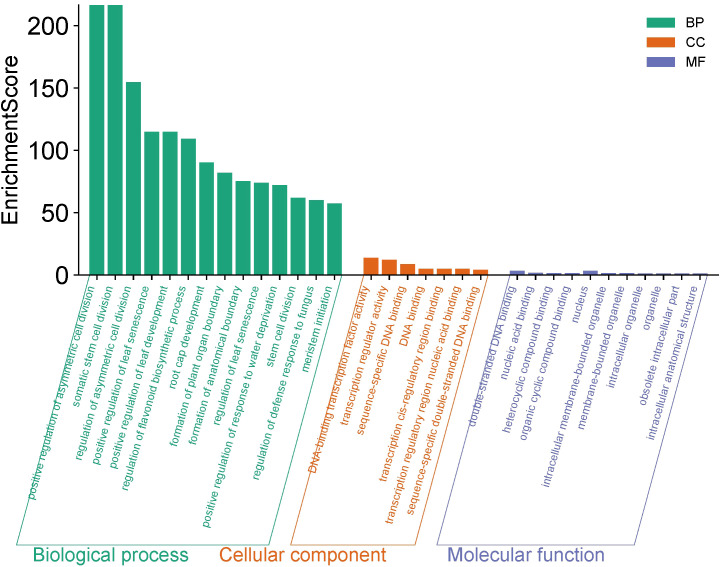
The GO annotation of *PoNAC* genes in ‘Fengdan’ peony. Biological process (BP) was marked in green, cellular component (CC) in red and molecular function (MF) in blue.

A total of 193 different GO annotation classes of *PoNAC* genes have been found (**Figure 4**), of which 11 are “molecular function” terms, mainly associated with the nucleus (nucleus), organelles (organelle, intracellular membrane-bounded organelle, intracellular anatomical structure, obsolete intracellular part, membrane-bounded organelle, and intracellular organelle) and binding (organic cyclic compound binding, heterocyclic compound binding, nucleic acid binding, and double-stranded DNA binding). On the other hand, the “cellular component” comprised 7 predominant categories mainly involved in DNA binding (DNA binding, DNA-binding transcription factor activity, sequence-specific DNA binding, and sequence-specific double-stranded DNA binding) and transcription regulation (transcription cis-regulatory region binding, transcription regulator activity, and transcription regulatory region nucleic acid binding). The remaining 175 classes belong to the subcategory “biological processes”, which is mainly related to cell division (positive regulation of asymmetric cell division, somatic stem cell division, regulation of asymmetric cell division, and stem cell division), organ development (positive regulation of leaf senescence, positive regulation of leaf development, root cap development, formation of plant organ boundary, formation of anatomical boundary, regulation of leaf senescence, and meristem initiation), flavonoid biosynthesis (regulation of flavonoid biosynthetic process), and stress responses (regulation of defense response to fungus, and positive regulation of response to water deprivation).

### Expression profiles of *PoNAC* genes in different tissues

**Figure 5:**
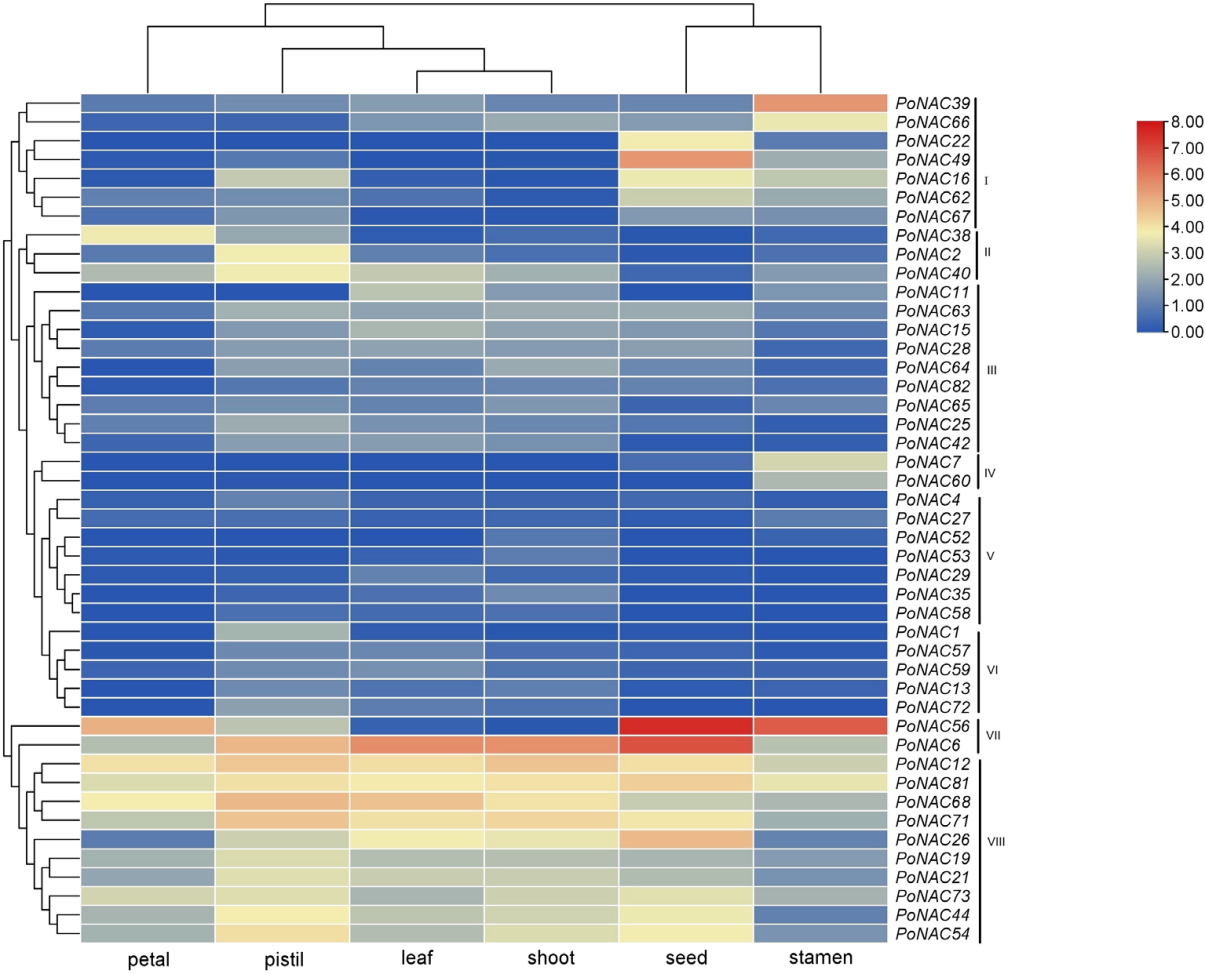
Expression profiles of PoNAC genes in 6 tissues of ‘Fengdan’ peony.

**Figure 5** presents the expression profiles of *PoNAC* genes investigated in 6 different tissues based on previous RNA-Seq data. The results showed the differential expression of only 45 in at least one tissue out of all 82 identified *PoNAC* genes. Cluster analysis of genes according to their expression profiles in different tissues revealed that they were divided into 8 groups, of which 4 groups(I, II, IV and VII) exhibited tissue specificity expression patterns, while 3 group (III, V, VI) were either not expressed in all tissues or expressed at extremely low levels, and 1 group (VIII)displayed expression in all tissues but with no obvious regularity of expression levels. 12 *PoNAC* genes, highly expressed in all tested tissues, included *PoNAC6*, *PoNAC12*, *PoNAC68*, etc., indicating that they may be the key regulators of plant growth and development. High levels of the expression of *PoNAC56* were observed in petals, whereas *PoNAC6*, *PoNAC12*, *PoNAC68*, and *PoNAC71* were highly expressed in pistils, and *PoNAC6* and *PoNAC68* were expressed at high levels in leaves. Furthermore, *PoNAC6* and *PoNAC12* achieved high expression levels in buds, while the high expression of *PoNAC48*, *PoNAC56*, *PoNAC6*, and *PoNAC26* was obtained in seeds, and *PoNAC39* and *PoNAC56* showed stamen-specific high expression, indicating their important roles in the development of different organs. All these tissue-specific *PoNAC* genes are considered potential targets for further studying their regulatory functions.

### Expression analysis of *PoNAC* genes in ‘Fengdan’ peony under abiotic stresses and ABA treatment

**Figure 6:**
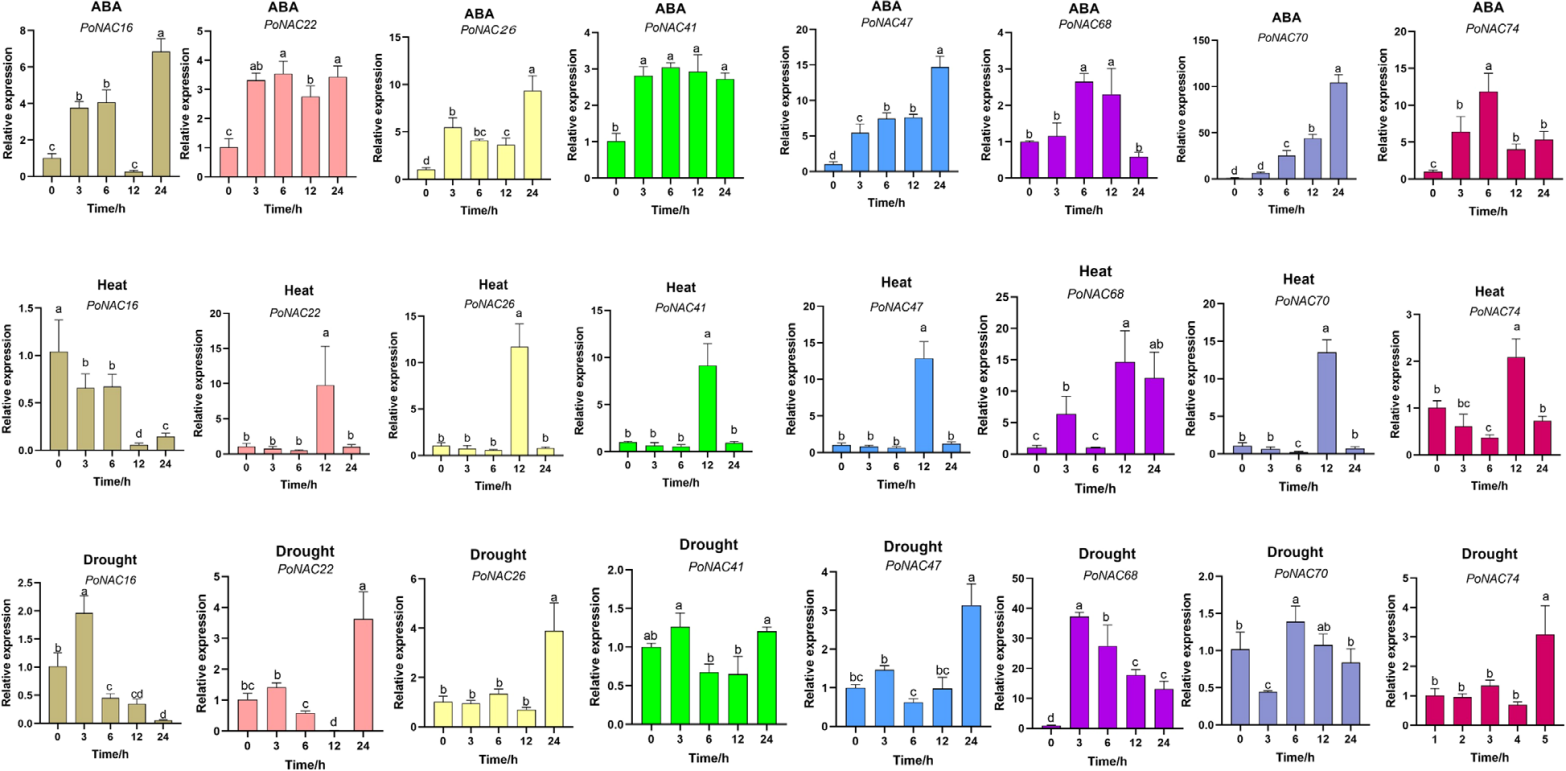
Expression levels of candidate PoNAC genes in the leaves of ‘Fengdan’ peony under different treatments. Error bars represent standard errors (SE) of three biological replicates. Different lowercase letters indicate statistically significant differences among means of treatments (P<0.05).

To investigate the expression patterns and potential functions of *PoNAC* genes, 8 *PoNAC* genes associated with stress responses from the ATAF and NAP subfamilies were selected, and their expression levels were detected under hormone treatment (ABA) and abiotic stresses (heat and drought) using RT-qPCR (**Figure 6**). Upon the ABA treatment, all these 8 *PoNAC* genes showed upregulation, with the maximum upregulation achieved for *PoNAC70* after 24 hours of the treatment, being 103 times higher than that observed for control. Except for a decrease in the expression level of *PoNAC16* between 0 and 12 hours of heat treatment, the peak of the expression level of other genes occurred at 12 h. A significant upregulation in the expression level of *PoNAC68* was noted, showing a 14-fold increase compared to that of the control group at 12 h. Under drought stress, the expression of *PoNAC26* and *PoNAC41* underwent little change within 24 h, while significant changes were recorded for other genes. A highly significant up-regulation trend was obtained for *PoNAC68* at 3 hours after drought stress, and its expression level was 37 times higher than that of the control group.

In conclusion, the *PoNAC* genes experienced significant upregulation of their expression under different treatments, and *PoNAC68* was likely a major regulatory gene for abiotic stress responses in tree peonies.

## Discussion

The NAC transcription factor family is of paramount importance in response to abiotic stresses (Nuruzzaman et al., 2013). NAC family members appear to have arisen in response to abiotic stresses in terrestrial plants during their transition from aquatic to terrestrial lifestyles (You et al., 2015) but have not yet been detected in multicellular algae. In this study, 82 *PoNAC* gene family members were identified based on the whole-genome data of tree peonies, which were higher in number than those found in *Scutellaria baicalensis* (56) (He et al., 2023), *Broussonetia papyrifera* (76) (Guo L. et al., 2024), and chickpea (72) (Singh et al., 2021), but lower than those in peanut (166) and pigeon bean (96) (Singh et al., 2021), *Arabidopsis* (117) and rice (151) (Ooak et al., 2003), oat (333) (Xu et al., 2024), and *salvia miltiorrhiza* (84) (Zhang et al., 2021). The variation in the number of NAC family members among species reflects the evolutionary divergence between plants.

A total of 19 pairs of *PoNAC* duplicated genes were identified, among which 14 pairs underwent WGD or segmental duplication, while 3 pairs were proximal duplicated genes, and 2 pairs were tandemly duplicated. It is speculated that all three types of duplication events, which have contributed to the expansion of the NAC family in ‘Fengdan’ peony, helping the plant to resist various stresses and adapt to constantly changing environments, mainly occurred in ONAC022, OSNAC7, and ANAC011 subfamilies. *PoNAC35* and *PoNAC72* in the OsNAC7 subfamily underwent WGD or segmental duplications with multiple copies, potentially driving the expansion of the *PoNAC* family. After gene duplication events, *PoNAC35* and *PoNAC72* retained their original function, while other gene copies could accumulate mutations without losing their functions, following which new gene regulatory networks arise and new functions and gene regulatory mechanisms evolve, which facilitate the better adaptation of ‘Fengdan’ peony to their environment (He and Zhang, 2005).

Each of these 82 PoNAC proteins, which were identified in ‘Fengdan’ peony in the present study and then categorized into 15 subfamilies, had their own unique functions. NAM subfamily members in Arabidopsis are crucial for floral organ development, regulating the flowering time, and meristem maintenance (Collinge and Boller, 2001). The high expression of *PoNAC56* in the NAM subfamily in petals, stamens, and seeds leads to the potential regulation of floral organ development and the formation of floral apical meristems in tree peonies. NAC proteins in ATAF, NAP, and OsNAC003 subfamilies may participate in stress response processes (Ooak et al., 2003). *PoNAC*s in these subfamilies, on the other hand, could perform vital functions in tree peonies, such as growth regulation and stress responses. *PoNAC68* in the ATAF subfamily is homologous to *ATAF1* (*ANAC2*), an NAC transcription factor in *Arabidopsis*. It is anticipated that besides response to biotic and abiotic stresses, *PoNAC68* has various fundamental roles in seed development, fruit maturation, promotion of leaf senescence and cotyledon opening, and enhancement of tolerance to salt stress in plants (Han et al., 2023).

Upon the exposure of plants to external stimuli, this information is transmitted to stress response systems through signal transduction pathways, which activate the plant’s resistance responses, thereby mitigating the damage caused by stress. *NAC* genes are of great importance in these processes and can be expressed in large quantities at high temperatures. For example, the expression of *SmNAC19* and *SmNAC75* in eggplant occurred at high levels after 3 h of heat stress (Wan et al., 2021), while the expression of NAC genes in ryegrass reached its peak at 24 or 48 hour post heat stress (Nie et al., 2020). NAC transcription factors improve plant tolerance to heat stress potentially through two pathways (the pathway of activation of HSF and HSPs, and the one of salicylic acid biosynthesis). In *Arabidopsis*, RCF2, the CBF gene regulator, transfers heat signal through dephosphorylation of *NAC019*, which subsequently binds to downstream of HSF promoters, leading to the activation of the expression of downstream of HSF and HSPs, and consequently helps the plant resist heat stress (Guan et al., 2014). *SIJA2* in tobacco could not only regulate the expression of the salicylic acid degradation gene but also reduce the accumulation of salicylic acid (SA), which may have been involved in heat stress response through the salicylic acid biosynthesis pathway (Liu et al., 2017). The variation of expression patterns of the selected 8 *PoNAC* genes occurred under heat stress, among which the expression of 6 *PoNAC* genes was rapidly induced within 0-12 hours, followed by a gradual decrease after 24 hours, while that of the remaining *PoNAC* genes underwent an initial decrease and then increased, indicating the quick response of peonies to heat stress. However, the regulatory mechanisms of *PoNAC* genes underlying heat stress response remain to be further investigated.

The gene duplication and functional diversification of NAC genes in plants during their evolution cause significant differences in their responses to stress. The ABA-dependent pathway is involved in drought stress response in plants (Yoshida et al., 2014). In Arabidopsis under drought stress, *AtNAC016* not only directly binds to the promoter of *AREB1* and causes the subsequent inhibition of its transcription but also directly targets *AtNAP*, which leads to the simultaneous regulation of *AtAREB1*. Although mutants of *AtNAC016* and *AtNAP* exhibited stronger drought resistance, reduced drought tolerance was observed in plant lines overexpressing them (Sakuraba et al., 2015). Meanwhile, apart from the direct activation of the transcription of the genes involved in GA inactivation (*SlGA2ox3*) and SA synthesis (*SlPAL3*), *SlNAP* also improved the drought resistance of tomatoes by activating the ABA synthesis gene *SlNCED1* (Wang et al., 2020). The increased drought resistance is also accompanied by reduced accumulation of reactive oxygen species (ROS) in plants. In rice, besides indirectly regulating PCD by activating *OsAP37* to stimulate caspase activity, *OsNAC2* suppresses *OsCOX11* to reduce ROS accumulation, which leads to enhanced drought resistance in the plant (Li et al., 2022). Under drought stress, *PoNAC26*, *PoNAC41*, and *PoNAC74*, which lacked the ABA-responsive element (ABRE) promoter, were expressed at lower levels, while the others containing the ABRE promoter exhibited higher expression levels, implying that the expression of *PoNAC* in ‘Fengdan’ peony may be controlled through the ABA-dependent pathway.

## Conclusions

In summary, a total of 82 PoNAC gene family members were identified from the genome of the ‘Fengdan’ peony and then classified into 15 subfamilies based on the phylogenetic analysis. *PoNAC* genes were relatively conserved while showing structural variation during the evolution. Additionally, duplication events occurred for 19 PoNAC gene pairs, which might drive the expansion of the NAC family in ‘Fengdan’ peony. The GO analysis results suggested that *PoNAC* genes were mostly concentrated in the “biological process” category. The differential expression patterns of 45 *PoNAC* genes in different tissues were observed, indicating their roles in the growth and development of some plant tissues. Meanwhile, the expressions of *PoNAC* genes were induced in response to the ABA treatment, and abiotic stresses. Overall, the systematic analysis of the NAC gene family in tree peonies contributed to our further study of the distinct functions of *PoNAC* genes, which lay a foundation for molecular breeding of tree peonies to enhance their stress resistance.

## Data Availability

The original contributions presented in the study are included in the article/**Supplementary Material**. Further inquiries can be directed to the corresponding authors.
