# Supplementary material for: Characterization of the NAC gene family in ‘Fengdan’ peony (Paeonia ostii) insights into the evolution and expression patterns under abiotic stresses and ABA treatment
Source: Front Plant Sci. 2025 Apr 16;16:1559667. doi: 10.3389/fpls.2025.1559667 (PMC12041088; doi:10.3389/fpls.2025.1559667)
Supplement: Supplementary file 2 [file Table1.docx]

**Supplementary Table 1 Primer sequences for *PoNAC* genes**

| Gene name | Primer sequence（5'→3'） | | |
| --- | --- | --- | --- |
|  | Forward |  | Reverse |
| *PoNAC16* | GAAGAACCCAGAATCAACCC | | CTTTATCTGTCCCAGTTGCC |
| *PoNAC22* | AGAGAGACCAGCTAATCGAG | | TCCAATTCGTTTTCACTCCC |
| *PoNAC26* | TTAAGGCTGGACGATTGGGT | | TGTGAGAAGGACGGAGATGG |
| *PoNAC41* | AACCGCAACCAGAACAACTC | | GTTACTTACGGCGCCTGTTT |
| *PoNAC47* | TCGGCCTATTCTCGCTTCAT | | ACTCGGCAGAGTACCCAATC |
| *PoNAC68* | GGTCAATTATCTCTGCCACC | | CGGGAACCATTTGGGTATTT |
| *PoNAC70* | ATCCAACAGACGAAGAGCTA | | TCCTCTCCATACAAAGCCAT |
| *PoNAC74* | ATGCACGAGTACAGGCTTCT | | GCGAGAGTAGATTGGGAGCA |
| *Ubiq*uitin | GAAGAACCCAGAATCAACCC | | TCAACGAATCTGTGAGATCGC |

**Supplementary Table. 2 Characteristics of NAC gene family members in ‘Fengdan’peony**

| Gene name | Gene number | Subcellular localization | Amino acid number/aa | Isoelectric point | Molecular weight/kDa | Grand Average  of Hydropathicity |
| --- | --- | --- | --- | --- | --- | --- |
| *PoNAC1* | Pos.gene1136.mRNA-1 | nucl | 422 | 5.86 | 48.01 | -0.676 |
| *PoNAC2* | Pos.gene2262.mRNA-1 | nucl | 289 | 6.62 | 33.27 | -0.647 |
| *PoNAC3* | Pos.gene2310.mRNA-1 | cyto | 198 | 8.84 | 23.14 | -0.415 |
| *PoNAC4* | Pos.gene4587.mRNA-1 | vacu | 360 | 8.87 | 41.00 | -0.537 |
| *PoNAC5* | Pos.gene7347.mRNA-1 | nucl | 726 | 5.61 | 83.09 | -0.664 |
| *PoNAC6* | Pos.gene8953.mRNA-1 | nucl | 263 | 8.99 | 29.86 | -0.713 |
| *PoNAC7* | Pos.gene9513.mRNA-1 | cyto | 709 | 8.57 | 81.06 | -0.817 |
| *PoNAC8* | Pos.gene10456.mRNA-1 | extr | 404 | 6.98 | 46.52 | -0.611 |
| *PoNAC9* | Pos.gene10746.mRNA-1 | nucl | 279 | 8.53 | 32.65 | -0.589 |
| *PoNAC10* | Pos.gene11876.mRNA-1 | nucl | 402 | 8.38 | 45.21 | -0.573 |
| *PoNAC11* | Pos.gene12171.mRNA-1 | nucl | 442 | 5.43 | 50.25 | -0.528 |
| *PoNAC12* | Pos.gene12208.mRNA-1 | chlo | 248 | 7.02 | 28.15 | -0.669 |
| *PoNAC13* | Pos.gene12244.mRNA-1 | nucl | 348 | 5.58 | 39.82 | -0.691 |
| *PoNAC14* | Pos.gene12345.mRNA-1 | vacu | 714 | 6.12 | 80.95 | -0.282 |
| *PoNAC15* | Pos.gene13285.mRNA-1 | chlo | 379 | 8.82 | 42.31 | -0.499 |
| *PoNAC16* | Pos.gene18498.mRNA-1 | nucl | 362 | 8.78 | 41.05 | -0.625 |
| *PoNAC17* | Pos.gene21391.mRNA-1 | mito | 165 | 8.36 | 19.61 | -0.956 |
| *PoNAC18* | Pos.gene21625.mRNA-1 | cyto | 410 | 5.15 | 47.23 | -0.761 |
| *PoNAC19* | Pos.gene22886.mRNA-1 | mito | 309 | 6.18 | 35.09 | -0.496 |
| *PoNAC20* | Pos.gene24999.mRNA-1 | nucl | 359 | 8.23 | 41.24 | -0.708 |
| *PoNAC21* | Pos.gene27799.mRNA-1 | chlo | 548 | 4.75 | 60.41 | -0.502 |
| *PoNAC22* | Pos.gene29870.mRNA-1 | nucl | 271 | 8.44 | 31.17 | -0.652 |
| *PoNAC23* | Pos.gene30398.mRNA-1 | nucl | 523 | 6.71 | 57.74 | -0.649 |
| *PoNAC24* | Pos.gene32085.mRNA-1 | nucl | 197 | 9.77 | 22.21 | -0.322 |
| *PoNAC25* | Pos.gene32449.mRNA-1 | chlo | 156 | 9.99 | 18.23 | -0.258 |
| *PoNAC26* | Pos.gene32451.mRNA-1 | nucl | 300 | 8.42 | 34.02 | -0.665 |
| *PoNAC27* | Pos.gene32658.mRNA-1 | nucl | 335 | 6.02 | 37.55 | -0.601 |
| *PoNAC28* | Pos.gene32905.mRNA-1 | cyto | 327 | 5.17 | 36.94 | -0.533 |
| *PoNAC29* | Pos.gene34729.mRNA-1 | chlo | 225 | 9.01 | 26.09 | -0.742 |
| *PoNAC30* | Pos.gene35708.mRNA-1 | nucl | 253 | 5.58 | 28.36 | -0.694 |
| *PoNAC31* | Pos.gene35710.mRNA-1 | golg | 122 | 6.97 | 14.13 | 0.145 |
| *PoNAC32* | Pos.gene37256.mRNA-1 | mito | 110 | 9.18 | 12.72 | -0.602 |
| *PoNAC33* | Pos.gene37553.mRNA-1 | nucl | 236 | 9.9 | 26.64 | -0.836 |
| *PoNAC34* | Pos.gene37996.mRNA-1 | pero | 380 | 6.55 | 43.48 | -0.874 |
| *PoNAC35* | Pos.gene39256.mRNA-1 | nucl | 360 | 6.32 | 41.06 | -0.784 |
| *PoNAC36* | Pos.gene39326.mRNA-1 | nucl | 151 | 5.67 | 17.87 | -0.664 |
| *PoNAC37* | Pos.gene39340.mRNA-1 | nucl | 277 | 9.71 | 31.80 | -0.861 |
| *PoNAC38* | Pos.gene39551.mRNA-1 | nucl | 374 | 6.46 | 42.40 | -0.564 |
| *PoNAC39* | Pos.gene40655.mRNA-1 | cyto | 223 | 6.12 | 25.18 | -0.304 |
| *PoNAC40* | Pos.gene41079.mRNA-1 | nucl | 236 | 9.6 | 27.11 | -0.572 |
| *PoNAC41* | Pos.gene41219.mRNA-1 | nucl | 271 | 8.92 | 31.06 | -0.688 |
| *PoNAC42* | Pos.gene41606.mRNA-1 | nucl | 463 | 6.31 | 52.51 | -0.965 |
| *PoNAC43* | Pos.gene42946.mRNA-1 | chlo | 330 | 9.16 | 37.83 | -0.571 |
| *PoNAC44* | Pos.gene43753.mRNA-1 | nucl | 626 | 4.88 | 70.25 | -0.674 |
| *PoNAC45* | Pos.gene44066.mRNA-1 | cysk | 310 | 8.1 | 35.84 | -0.793 |
| *PoNAC46* | Pos.gene44911.mRNA-1 | cyto | 400 | 5.35 | 46.23 | -0.744 |
| *PoNAC47* | Pos.gene45075.mRNA-1 | chlo | 333 | 9.23 | 38.46 | -0.689 |
| *PoNAC48* | Pos.gene45080.mRNA-1 | nucl | 390 | 6.25 | 43.89 | -0.61 |
| *PoNAC49* | Pos.gene46554.mRNA-1 | nucl | 374 | 8.12 | 41.16 | -0.556 |
| *PoNAC50* | Pos.gene46630.mRNA-1 | chlo | 212 | 6.83 | 24.77 | -0.809 |
| *PoNAC51* | Pos.gene46667.mRNA-1 | nucl | 120 | 8.82 | 14.14 | -0.758 |
| *PoNAC52* | Pos.gene46710.mRNA-1 | nucl | 303 | 5.18 | 34.28 | -0.733 |
| *PoNAC53* | Pos.gene47723.mRNA-1 | cyto | 572 | 9.12 | 65.38 | -0.547 |
| *PoNAC54* | Pos.gene48249.mRNA-1 | nucl | 429 | 5.09 | 48.45 | -0.843 |
| *PoNAC55* | Pos.gene50239.mRNA-1 | cyto | 207 | 9 | 23.62 | -0.586 |
| *PoNAC56* | Pos.gene51093.mRNA-1 | nucl | 324 | 8.28 | 36.10 | -0.712 |
| *PoNAC57* | Pos.gene52748.mRNA-1 | nucl | 369 | 4.96 | 41.91 | -0.673 |
| *PoNAC58* | Pos.gene53615.mRNA-1 | nucl | 373 | 6.32 | 43.05 | -0.881 |
| *PoNAC59* | Pos.gene56382.mRNA-1 | nucl | 368 | 9.32 | 42.08 | -0.265 |
| *PoNAC60* | Pos.gene56736.mRNA-1 | cyto | 387 | 6.11 | 45.13 | -0.895 |
| *PoNAC61* | Pos.gene57377.mRNA-1 | cyto | 316 | 5.3 | 36.39 | -0.698 |
| *PoNAC62* | Pos.gene58812.mRNA-1 | nucl | 442 | 7.26 | 50.47 | -0.789 |
| *PoNAC63* | Pos.gene59602.mRNA-1 | cyto | 328 | 5.39 | 37.28 | -0.489 |
| *PoNAC64* | Pos.gene59666.mRNA-1 | nucl | 444 | 5.19 | 49.47 | -0.708 |
| *PoNAC65* | Pos.gene60528.mRNA-1 | nucl | 536 | 4.86 | 60.64 | -0.71 |
| *PoNAC66* | Pos.gene63853.mRNA-1 | nucl | 297 | 8.31 | 33.42 | -0.416 |
| *PoNAC67* | Pos.gene63916.mRNA-1 | cyto | 281 | 5.6 | 31.89 | -0.583 |
| *PoNAC68* | Pos.gene66003.mRNA-1 | pero | 159 | 9.82 | 18.15 | -0.7 |
| *PoNAC69* | Pos.gene66588.mRNA-1 | nucl | 320 | 8.36 | 36.24 | -0.62 |
| *PoNAC70* | Pos.gene66898.mRNA-1 | cyto | 305 | 8.38 | 34.96 | -0.47 |
| *PoNAC71* | Pos.gene66948.mRNA-1 | nucl | 720 | 5.26 | 81.06 | -0.635 |
| *PoNAC72* | Pos.gene67223.mRNA-1 | nucl | 236 | 5.42 | 27.36 | -0.787 |
| *PoNAC73* | Pos.gene70140.mRNA-1 | cyto | 555 | 5.04 | 62.93 | -0.584 |
| *PoNAC74* | Pos.gene72400.mRNA-1 | chlo | 319 | 9.3 | 35.82 | -0.497 |
| *PoNAC75* | Pos.gene73832.mRNA-1 | nucl | 273 | 9.77 | 31.51 | -0.432 |
| *PoNAC76* | Pos.gene75152.mRNA-1 | nucl | 353 | 4.97 | 40.23 | -0.69 |
| *PoNAC77* | Pos.gene75190.mRNA-1 | nucl | 147 | 9.11 | 17.03 | -0.822 |
| *PoNAC78* | Pos.gene76000.mRNA-1 | nucl | 548 | 5.61 | 62.99 | -0.82 |
| *PoNAC79* | Pos.gene80266.mRNA-1 | cyto | 279 | 4.94 | 32.53 | -0.752 |
| *PoNAC80* | Pos.gene80487.mRNA-1 | golg | 224 | 9.09 | 26.48 | -0.063 |
| *PoNAC81* | Pos.gene80994.mRNA-1 | chlo | 447 | 6.2 | 50.69 | -0.781 |
| *PoNAC82* | Pos.gene83286.mRNA-1 | nucl | 524 | 6.59 | 57.70 | -0.637 |

Note：nucl for nucleus; chol for chloroplast; cyto for cytoplasm; extr for extracellular space; mito for mitochondria; golg for Golgi Apparatus; pero for peroxisome; cysk for cytoskeleton; vacu for vacuole.

**Supplementary Table. 3 The Ka, Ks, and Ka/Ks values of *PoNAC* gene pairs derived from different duplication types**

| Gene pair | Ka | Ks | Ka/Ks | Duplication type |
| --- | --- | --- | --- | --- |
| PoNAC13-PoNAC67 | 0.696118 | 3.87527 | 0.179631 | WGD or segmental duplication |
| PoNAC20-PoNAC8 | 0.717746 | 3.82728 | 0.187534 | WGD or segmental duplication |
| PoNAC22-PoNAC40 | 0.599936 | 3.75518 | 0.159762 | WGD or segmental duplication |
| PoNAC24-PoNAC35 | 0.7391 | 3.55233 | 0.208061 | WGD or segmental duplication |
| PoNAC25-PoNAC26 | 0.371827 | 0.622976 | 0.596856 | Proximal duplication |
| PoNAC30-PoNAC52 | 0.0965 | 0.171133 | 0.563887 | Proximal duplication |
| PoNAC31-PoNAC37 | 0.376088 | 0.963903 | 0.390172 | tandem duplication |
| PoNAC33-PoNAC35 | 0.756386 | 3.66031 | 0.206645 | WGD or segmental duplication |
| PoNAC41-PoNAC70 | 0.0116802 | 0.00966144 | 1.20894 | Proximal duplication |
| PoNAC42-PoNAC49 | 0.863955 | 4.02808 | 0.214483 | WGD or segmental duplication |
| PoNAC44-PoNAC53 | 0.794102 | 3.98093 | 0.199477 | WGD or segmental duplication |
| PoNAC49-PoNAC24 | 0.709731 | 2.88091 | 0.246357 | WGD or segmental duplication |
| PoNAC62-PoNAC33 | 0.663913 | 3.54327 | 0.187373 | WGD or segmental duplication |
| PoNAC72-PoNAC51 | 0.459107 | 3.10103 | 0.14805 | WGD or segmental duplication |
| PoNAC72-PoNAC64 | 0.865125 | 3.66704 | 0.235919 | WGD or segmental duplication |
| PoNAC72-PoNAC66 | 0.571944 | 3.70186 | 0.154502 | WGD or segmental duplication |
| PoNAC75-PoNAC49 | 0.701923 | 3.86904 | 0.181421 | WGD or segmental duplication |
| PoNAC81-PoNAC12 | 0.195152 | 1.08801 | 0.179367 | tandem duplication |
| PoNAC9-PoNAC11 | 0.740736 | 3.8289 | 0.193459 | WGD or segmental duplication |

**Supplementary Table 4 Classification table of NAC family**

| PoNAC50 |  |
| --- | --- |
| ANAC24 |  |
| ANAC23 |  |
| PoNAC23 |  |
| PoNAC82 |  |
| PoNAC39 |  |
| PONAC27 |  |
| PoNAC65 |  |
| PoNAC77 |  |
| PoNAC28 | ONAC003 |
| PoNAC14 |  |
| PoNAC54 |  |
| ANAC８ |  |
| PoNAC64 |  |
| ANAC85 |  |
| ANAC44 |  |
| ANAC99 |  |
| PoNAC42 |  |
| ANAC75 |  |
| ANAC73 |  |
| PoNAC20 |  |
| PoNAC4 |  |
| ANAC10 |  |
| ANAC27 |  |
| ANAC88 |  |
| ANAC6 |  |
| PoNAC79 |  |
| PoNAC78 |  |
| PONAC46 |  |
| PoNAC18 |  |
| ANAC64 | ANAC063 |
| ANAC93 |  |
| ANAC65 |  |
| ANAC63 |  |
| PoNAC13 |  |
| ANAC25 | NAP |
| PoNAC56 |  |
| ANAC56 |  |
| ANAC18 |  |
| PoNAC74 |  |
| PoNAC47 |  |
| PoNAC16 |  |
| PoNAC43 |  |
| ANAC29 |  |
| ANAC47 | AtNAC3 |
| PoNAC7 |  |
| PoNAC60 |  |
| PoNAC25 |  |
| ANAC72 |  |
| ANAC55 |  |
| ANAC19 |  |
| PoNAC70 | ATAF |
| PoNAC41 |  |
| PoNAC22 |  |
| ANAC102 |  |
| ANAC81 |  |
| ANAC32 |  |
| PoNAC26 |  |
| PoNAC68 |  |
| ANAC2 |  |
| PoNAC19 |  |
| ANAC104 |  |
| ANAC95 |  |
| PoNAC53 | ONAC022 |
| PoNAC80 |  |
| PoNAC45 |  |
| ANAC42 |  |
| ANAC94 |  |
| ANAC9 |  |
| PoNAC33 |  |
| PoNAC75 |  |
| PoNAC48 |  |
| PoNAC10 |  |
| PoNAC11 |  |
| ANAC35 |  |
| ANAC34 |  |
| ANAC36 |  |
| PoNAC2 |  |
| PoNAC3 |  |
| PoNAC24 |  |
| PoNAC51 |  |
| PoNAC32 |  |
| PoNAC55 |  |
| ANAC97 | ANAC001 |
| ANAC5 |  |
| ANAC4 |  |
| ANAC48 |  |
| ANAC3 |  |
| ANAC49 |  |
| ANAC1 |  |
| ANAC69 |  |
| ANAC68 |  |
| ANAC67 |  |
| ANAC90 | TERN |
| ANAC61 |  |
| PoNAC37 |  |
| PoNAC30 |  |
| PoNAC52 |  |
| PoNAC31 | OsNAC8 |
| ANAC77 |  |
| PoNAC15 |  |
| ANAC40 |  |
| ANAC89 |  |
| ANAC60 |  |
| PoNAC71 | TIP |
| ANAC91 |  |
| ANAC62 |  |
| PoNAC44 |  |
| PoNAC14 |  |
| ANAC13 | NAC2 |
| PoNAC73 |  |
| ANAC17 |  |
| ANAC16 |  |
| PoNAC63 |  |
| PoNAC21 |  |
| ANAC103 |  |
| ANAC82 |  |
| ANAC50 |  |
| ANAC52 |  |
| ANAC51 |  |
| PoNAC12 |  |
| PoNAC81 |  |
| ANAC78 |  |
| ANAC53 |  |
| PoNAC76 | ANAC011 |
| ANAC20 |  |
| PoNAC5 |  |
| ANAC28 |  |
| PoNAC72 |  |
| ANAC57 |  |
| ANAC86 |  |
| ANAC45 |  |
| PoNAC29 |  |
| ANAC11 |  |
| ANAC96 |  |
| ANAC71 |  |
| PoNAC61 |  |
| PoNAC59 |  |
| PoNAC9 |  |
| PoNAC57 |  |
| PoNAC40 | SENU5 |
| ANAC84 |  |
| ANAC41 |  |
| PoNAC6 |  |
| ANAC83 |  |
| ANAC66 | OsNAC7 |
| ANAC43 |  |
| PoNAC1 |  |
| ANAC12 |  |
| PoNAC8 |  |
| ANAC33 |  |
| ANAC15 |  |
| PoNAC34 |  |
| ANAC70 |  |
| ANAC101 |  |
| ANAC105 |  |
| ANAC30 |  |
| PoNAC35 |  |
| PoNAC36 |  |
| ANAC76 |  |
| ANAC37 |  |
| ANAC26 |  |
| ANAC7 |  |
| PoNAC17 |  |
| PoNAC58 |  |
| PoNAC67 | NAC1 |
| ANAC74 |  |
| PoNAC66 |  |
| ANAC22 |  |
| ANAC21 |  |
| PoNAC38 | NAM |
| ANAC31 |  |
| ANAC98 |  |
| PoNAC49 |  |
| ANAC54 |  |
| PoNAC69 |  |
| ANAC58 |  |
| ANAC39 |  |
| ANAC38 |  |
| PoNAC62 |  |
| ANAC87 |  |
| ANAC46 |  |
| ANAC92 |  |
| ANAC59 |  |
| ANAC100 |  |
| ANAC80 |  |
| ANAC79 |  |

**Supplementary Table 5 The data for figure 5**

| gene_id | leaf | petal | pistil | seed | shoot | stamen |
| --- | --- | --- | --- | --- | --- | --- |
| PoNAC1 | 0.13 | 0 | 4.04 | 0.05 | 0.04 | 0 |
| PoNAC2 | 1.08 | 0.85 | 12.36 | 0.05 | 0.57 | 0.6 |
| PoNAC4 | 0.31 | 0.22 | 1.15 | 0.41 | 0.31 | 0.16 |
| PoNAC6 | 49.11 | 4.93 | 27.67 | 112.07 | 48.05 | 5.33 |
| PoNAC7 | 0 | 0 | 0 | 0.51 | 0 | 7.98 |
| PoNAC11 | 5.55 | 0 | 0 | 0 | 2.31 | 1.99 |
| PoNAC12 | 16.3 | 15.66 | 22.08 | 15.89 | 23.22 | 7.17 |
| PoNAC13 | 0.65 | 0 | 1.46 | 0.1 | 1.03 | 0.28 |
| PoNAC15 | 4.29 | 0.1 | 2.21 | 2.14 | 2.83 | 0.76 |
| PoNAC16 | 0.17 | 0.08 | 6.34 | 11.57 | 0.04 | 6.01 |
| PoNAC19 | 4.98 | 3.89 | 8.94 | 4.15 | 5.11 | 2.31 |
| PoNAC21 | 6.8 | 2.92 | 9.44 | 4.83 | 6.83 | 1.79 |
| PoNAC22 | 0 | 0 | 0 | 12.42 | 0 | 0.9 |
| PoNAC25 | 1.8 | 1.06 | 3.4 | 0.77 | 1.35 | 0.16 |
| PoNAC26 | 12.46 | 0.91 | 7.37 | 26.47 | 10.81 | 1.18 |
| PoNAC27 | 0.25 | 0.47 | 0.55 | 0.1 | 0.35 | 0.94 |
| PoNAC28 | 2.73 | 0.92 | 2.34 | 2.51 | 2.32 | 0.35 |
| PoNAC29 | 1.15 | 0.07 | 0.2 | 0.06 | 0.39 | 0 |
| PoNAC35 | 0.61 | 0 | 0.34 | 0 | 1.47 | 0 |
| PoNAC38 | 0.11 | 12.02 | 3.11 | 0.04 | 0.49 | 0.37 |
| PoNAC39 | 2.38 | 0.93 | 1.55 | 1.32 | 1.32 | 43.69 |
| PoNAC40 | 6.14 | 4.69 | 12.11 | 0.35 | 3.77 | 2.23 |
| PoNAC42 | 2.35 | 0.33 | 2.42 | 0.07 | 1.75 | 0.17 |
| PoNAC44 | 5.76 | 4.11 | 12.65 | 11.6 | 7.72 | 1.12 |
| PoNAC49 | 0 | 0.07 | 0.8 | 43.53 | 0.04 | 3.51 |
| PoNAC52 | 0 | 0 | 0 | 0 | 0.78 | 0.27 |
| PoNAC53 | 0.26 | 0.06 | 0.05 | 0 | 0.97 | 0 |
| PoNAC54 | 4.87 | 3.87 | 16.18 | 12.39 | 8.86 | 1.85 |
| PoNAC56 | 0.22 | 30.82 | 5.68 | 187.92 | 0.04 | 97.09 |
| PoNAC57 | 1.33 | 0.04 | 1.45 | 0.29 | 0.51 | 0.07 |
| PoNAC58 | 0.45 | 0 | 0.56 | 0 | 0.61 | 0 |
| PoNAC59 | 1.69 | 0.29 | 1.5 | 0.32 | 0.69 | 0.32 |
| PoNAC60 | 0 | 0 | 0.05 | 0 | 0 | 4.46 |
| PoNAC62 | 0.64 | 1.11 | 1.56 | 6.92 | 0.08 | 3.28 |
| PoNAC63 | 2.74 | 0.8 | 3.8 | 3.26 | 3.41 | 1.32 |
| PoNAC64 | 1.19 | 0 | 2.5 | 1.37 | 3.24 | 0.33 |
| PoNAC65 | 1.19 | 0.97 | 1.64 | 0.32 | 2.07 | 1.31 |
| PoNAC66 | 1.99 | 0.29 | 0.33 | 2.32 | 3.27 | 11.3 |
| PoNAC67 | 0.05 | 0.61 | 2.08 | 2.2 | 0.05 | 1.77 |
| PoNAC68 | 23.79 | 12.57 | 27.34 | 6.62 | 15.03 | 4.39 |
| PoNAC71 | 15.61 | 6.03 | 23.12 | 14.27 | 17.9 | 3.61 |
| PoNAC72 | 0.96 | 0 | 2.54 | 0 | 0.65 | 0 |
| PoNAC73 | 4.17 | 7.92 | 9.25 | 9.74 | 7.31 | 3.92 |
| PoNAC81 | 13.44 | 8.9 | 15.45 | 19.93 | 15.33 | 10.68 |
| PoNAC82 | 1.19 | 0.07 | 0.76 | 1.19 | 1.29 | 0.6 |
